# Targeting met mediated epithelial-mesenchymal transition in the treatment of breast cancer

**DOI:** 10.1186/s40169-014-0030-5

**Published:** 2014-09-26

**Authors:** Paul W Sylvester

**Affiliations:** School of Pharmacy, University of Louisiana at Monroe, 700 University Avenue, Monroe, 71209-0470 LA USA

**Keywords:** Met, HGF, Epithelial mesenchymal transition, Targeted therapy, cancer, tocotrienols

## Abstract

Mesenchymal epithelial transition factor receptor (Met) is a receptor tyrosine kinase that plays a critical role in promoting cancer cell malignant progression. Met is activated by its ligand hepatocyte growth factor (HGF). HGF-dependent Met activation plays an important role in stimulating epithelial-mesenchymal transition (EMT) in tumor cells, resulting in increased tumor cell proliferation, survival, motility, angiogenesis, invasion, and metastasis. The HGF/Met axis has thus attracted great interest as a potential target in the development of novel cancer therapies. In an effort to suppress tumor cell malignant progression, efforts have been made to develop agents capable of inhibiting inhibit Met-induced EMT, including specific Met tyrosine kinase inhibitors, HGF antagonists that interfere with HGF binding to Met, and antibodies that prevent Met activation and/or dimerization. Tocotrienols, a subgroup within the vitamin E family of compounds, display potent anticancer activity that results, at least in part, from inhibition of HGF-dependent Met activation and signaling. The present review will provide a brief summary of the increasing importance of the HGF/Met axis as an attractive target for cancer chemotherapy and the role of tocotrienols in suppressing Met activation, signaling and HGF-induced EMT in breast cancer cells. Evidence provided suggests that γ-tocotrienol therapy may afford significant benefit in the treatment of breast cancers characterized by Met dysregulation.

**Electronic supplementary material:**

The online version of this article (doi:10.1186/s40169-014-0030-5) contains supplementary material, which is available to authorized users.

## Introduction

Traditional cancer chemotherapeutic agents typically exhibit non-selective activity and often produce low response rates and severe toxic side effects in patients [1]. Thus, there is currently great interest in developing novel therapeutic agents that target signaling molecules involved in the growth, survival and progression of malignant cells with high specificity. The advantage of such targeted therapies is enhanced efficacy and a reduction in adverse side effects. A promising intracellular target is the receptor tyrosine kinase, mesenchymal epithelial transition factor (Met). Expressed in cells of epithelial origin, Met is activated by its ligand hepatocyte growth factor (HGF; scatter factor), which is produced by mesenchymal cells [2],[3]. HGF/Met signaling is critical for epithelial-mesenchymal interactions underlying normal functions including cell migration, morphology, cell division during tissue remodeling and repair, and organogenesis during embryonic development [3],[4]. However, Met is also implicated in malignant progression. Excessive or unregulated tumor cell Met activity is associated with poor patient prognosis owing to aggressive malignant phenotype typified by enhanced angiogenesis, invasion and metastasis [5]-[8].

HGF-dependent activation of Met leads to receptor dimerization, tyrosine autophosphorylation, and initiation of down-stream signaling, including activation of MAPK, PI3K/Akt, and STAT pathways, all of which play an important role in cancer cell proliferation and survival [7],[9],[10]. However, oncogenic mutations can result in Met overexpression and/or aberrant signaling that can lead to epithelial-to-mesenchymal transition (EMT) [8],[11],[12]. Epithelial cells that undergo EMT lose epithelial cell characteristics and acquire a mesenchymal phenotype characterized by migratory and invasive characteristics [9],[12],[13]. Epithelial and mesenchymal cells differ regarding function, morphology and expression of phenotype-specific protein cell markers. Epithelial cells express high levels of cytokeratin, E-cadherin, and β-catenin proteins, whereas N-cadherin, fibronectin and vimentin proteins occur at high levels in mesenchymal cells [6],[8],[11]-[18]. Moreover, cells undergoing EMT display cancer stem cell-like properties that are associated with enhanced malignant potential and progression [19].

Tumors exhibiting rapid growth often face insufficient blood flow to meet their high oxygen demands, but are able to adapt to hypoxic conditions by altering metabolism and phenotypic functions [20]. A typical compensatory response to hypoxia is increased production of hypoxia-inducible factor-1 (HIF-1), which reportedly increases HGF-dependent MET expression and signaling [21],[22]. Since cancer cell invasion and metastasis are primary causes of cancer patient death, therapeutic strategies that target and suppress malignant progression may significantly improve patient survival. Thus, HGF-dependent Met activation has emerged as an attractive target for therapeutic intervention because of its role in promoting tumor cell EMT, angiogenesis, proliferation and survival [23].

Various approaches have been used to inhibit Met activation and signaling, including agents that inhibit tyrosine kinase activity (K252a, SUii274, PHA-665752, and PF2341066), neutralizing antibodies (anti-HGF antibodies) and competitive antagonists (NK4 and uncleavable HGF) that interfere with HGF binding and activation of Met, and agents that block HGF binding to Met and/or Met receptor dimerization (anti-Met antibodies) [4]. Recent investigations also show that certain natural products display potent inhibitory effects against Met activation and signaling. Tocotrienol, a rare natural form of vitamin E, is one such natural product that potently inhibits Met activation and signaling and displays potent anticancer activity at treatment doses that have minimal or no effect on normal cell function or viability [24],[25]. The present review will present a brief summary of evidence supporting the HGF/Met axis as a plausible target for cancer chemotherapy, and the role of tocotrienols in suppressing Met activation, signaling and HGF-induced EMT.

## Review

### Met activation and signaling

Met is expressed exclusively in epithelial cells, whereas its natural ligand HGF is produced solely by mesenchymal cells [3],[4],[26]-[29]. Mediated by paracrine mechanisms [30], HGF activation of Met is intimately involved in epithelial cell phenotypic morphology and behavior, including cell proliferation, motility, invasion, angiogenesis, and branching tubulogenesis [9],[10],[31]-[33]. All these events occur during normal events such as embryogenesis, and tissue remodeling, regeneration, and repair in adults [13],[26],[34].

Different biological effects of Met signaling are directly associated with activation of specific intracellular pathways. HGF-induced cell proliferation is mediated by activation of the MAPK cascade [35], whereas activation of the PI3K/Akt pathway promotes cell survival and prevents caspase-dependent apoptosis [9],[10],[36]. The ability of HGF to induce epithelial cell morphological changes and motility requires disruption of cell-to-cell adhesion and dissociation of the basal lamina from the extracellular matrix [13],[37]. HGF-induced cell scattering and invasion is achieved by activation of several different signaling molecules and pathways, including PI3K, Rac and Rho [13],[37]. Additionally, Met activation can stimulate vascular endothelial growth factor (VEGF) production to enhance angiogenesis [10],[26]. Met forms heterodimers with other receptor tyrosine kinases including members of the epidermal growth factor (EGF) receptor family; this crosstalk between Met and other growth factor receptors permits integration of signals during the process of phenotypic change [38].

Met mutations, overexpression and/or dysregulation commonly occur in a wide variety of cancers, characteristics that are associated with promotion of cancer progression, including tumor cell proliferation, survival, motility, EMT, angiogenesis, invasion and metastasis [9],[10],[39]. The majority of mutations that result in constitutive activation of Met occur in the receptor's cytoplasmic tyrosine kinase domain [31], while mutations in the autoregulatory domain of the receptor allow for persistent and unattenuated responsiveness to HGF stimulation [13],[35]. Overexpression of HGF can also induce oncogenic effects through over-stimulation of the non-mutated Met receptor, which can lead to enhanced tumor cell progression and malignant phenotype [10],[40],[41]. Enhanced HGF expression is specifically implicated as a promoter and risk factor in breast cancer [42]. Previous studies show that elevated circulating HGF levels correlate with lower survival rates and increased risk of metastasis in breast cancer patients [43],[44]. Occurrence of high levels of HGF in breast cancer biopsies is associated with an aggressive malignant phenotype [5]. Studies show that Met is overexpressed in nearly 30% of all breast cancers and is a strong independent predictor of poor patient prognosis and survival [30],[43]. Microarray analysis of tissue obtained from breast cancer patients shows that elevations in Met expression occur in a significant percentage of EGFR/HER2 negative tumors [30].

### Inhibitors of HGF-induced Met activation and signaling

**Figure 1:**
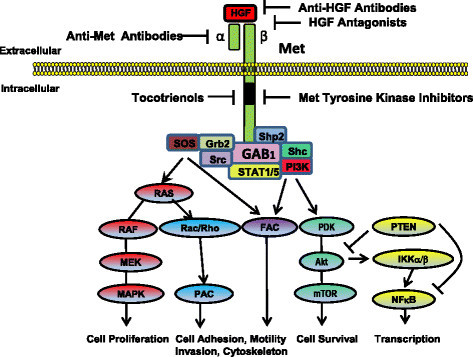
***Schematic representation of the Met receptor and therapeutic strategies currently being developed to inhibit HGF-dependent Met activation and signaling in cancer.*** The Met receptor has an extracellular α-chain that binds HGF and a transmembrane β-chain that contains the tyrosine kinase domain and autophosphorylation sites that are essential for interacting with substrates. Activation of Met by HGF leads to receptor dimerization and recruitment of adaptor (GAB1, Grb2, Shc) and signaling (Ras/MAPK, PI3K/Akt, Src, STAT, Shp2) proteins. Downstream signaling promotes cell proliferation, altered cytoskeletal function, decreased cellular adhesion, increased cellular invasion, decreased apoptosis and enhanced DNA transcription. Anti-HGF approaches to inhibit Met signaling include anti-HGF antibodies that neutralize HGF and antagonists that block HGF binding to the Met receptor. A second approach includes the use of anti-Met antibodies that prevent HGF binding to Met or Met dimerization. Another approach is the use of specific Met tyrosine kinase inhibitors that prevent receptor second messenger signaling. Tocotrienols have also been found to be potent inhibitors of Met activation and signaling, but the exact mechanism mediating these effects are not completely understood at present. Targeting aberrant Met signaling in cancer cells can inhibit of downstream signaling pathways involved with tumor cell proliferation, motility, viability, morphology and epithelial-to-mesenchymal transition.

Since Met plays an important role in malignant progression, it has emerged as a practical target in cancer chemotherapy, with various strategies currently under development to inhibit Met activation and signaling. Agents that decrease receptor tyrosine kinase activity include low molecular weight molecules that block ATP binding to Met's catalytic, thereby inhibiting receptor autophosphorylation and recruitment of molecules associated with downstream signaling [4]. Several Met tyrosine kinase inhibitors, including K252a, SU11274, and PHA-665752, have been developed and studied extensively [4]. While these agents are reported to be very effective in suppressing kinase activity, their clinical usefulness is somewhat limited because they do not inhibit Met transcription and translation [45]. Subsequently, a second generation of agents developed in recent years are currently being tested in clinical trials. The use of Met tyrosine kinase inhibitors and other Met inhibitors in the treatment of human cancers has been recently reviewed in detail [46]. The inhibitory actions of these agents are summarized in Figure 1.

Agents that inhibit HGF include NK4, anti-HGF neutralizing antibodies, and an uncleavable HGF agonist. NK4 is a HGF-like ligand that binds to Met without activating the receptor [47], whereas the neutralizing anti-HGF antibodies act on various regions of the HGF molecule to prevent HGF binding to and activation of Met [48]. The uncleavable form of HGF is not biologically active, but interacts with the ligand binding site on Met to block receptor activation [49],[50]. However, HGF inhibitors have also been found to have somewhat limited use because they only suppress HGF-dependent Met activation and are not effective against mutated Met receptors that are constitutively active (4).

### Tocotrienol inhibition of HGF-induced Met activation and epithelial-mesenchymal transition

**Figure 2:**
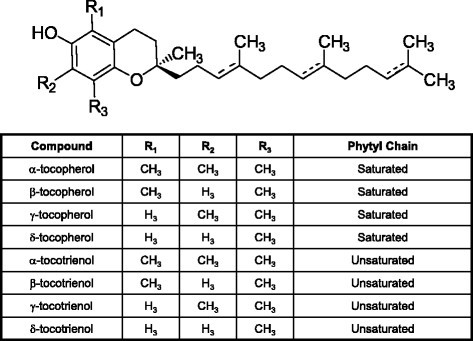
***Generalized chemical structure of natural tocopherols and tocotrienols that make up the vitamin E family of compounds.***

Vitamin E represents a family of compounds that is divided into structurally similar tocopherol and tocotrienol subgroups [51],[52]. These subgroups differ as tocopherols have a saturated, whereas tocotrienols have an unsaturated phytyl chain attached to a chromane ring structure [51],[52], as shown in Figure 2. However, only tocotrienols displays potent anticancer activity at treatment doses that do not affect normal cell growth or viability [53],[54]. Individual isoforms (α, β, γ, and δ) of tocopherols and tocotrienols are differentiated by degree of chromane ring methylation (Figure 2). Previous studies show that antiproliferative and apoptotic effects of tocotrienols are mediated, at least in part, by their ability to inhibit EGF receptor family member activation and signal transduction [55]-[57]. γ-Tocotrienol inhibition of mammary tumor cell growth is mediated by suppression of receptor tyrosine kinase activity of HER3/ErbB3, HER4/ErbB4, and to a lesser extent HER2/ErbB2, but not HER1/ErbB1, and attenuation of receptor downstream pathways that include MAPK, PI3K/Akt, STAT, and NFκB signaling [55]-[57]. Subsequent work demonstrated that γ-tocotrienol is also a powerful inhibitor of HGF-induced Met tyrosine kinase activation and signaling [24],[25].

Studies using + SA mammary tumor cells maintained in serum-free defined media containing HGF as a mitogen show that HGF induces a dose-responsive increase in growth and corresponding increase in Met autophosphorylation, whereas combined treatment with γ-tocotrienol blocks these effects [24]. In addition, γ-tocotrienol treatment is reported to significantly reduce total Met levels in these cells. In contrast, similar treatment with growth-inhibiting doses of the Met tyrosine kinase inhibitor SU11274 inhibits HGF-dependent Met autophosphorylation, but has no effect on cellular levels of Met [24]. Similarly, tumor cells transfection with Met specific siRNA significantly inhibits HGF-dependent growth [24].

Subsequent studies confirm and extend these findings in human breast cancer cell lines [25]. Results show that treatment with either γ-tocotrienol or the Met tyrosine kinase inhibitor SU11274 alone induces dose-dependent inhibition in estrogen receptor- positive MCF-7 and estrogen receptor negative MDA-MB 231 human breast cancer cells. However, combined treatment with subeffective doses (non-growth inhibiting) of γ-tocotrienol and SU11274 results in synergistic inhibition of MCF-7 and MDA-MB-231 cancer cells, while these same treatments have no effect on growth or viability of immortalized normal MCF10A human mammary epithelial cells [25]. These finding demonstrate that tocotrienol treatment alone or in combination with Met inhibitors selectively inhibits growth of breast cancer cells without affecting normal cell growth or viability. In addition, combination treatment significantly reduces Met autophosphorylation and phosphorylation (activation) of receptor substrates involved in downstream signal transduction, including STAT1, STAT5, Akt, and NFκB (26), and significantly inhibits breast cancer cell motility and migration as determined by wound healing assay [25].

**Figure 3:**
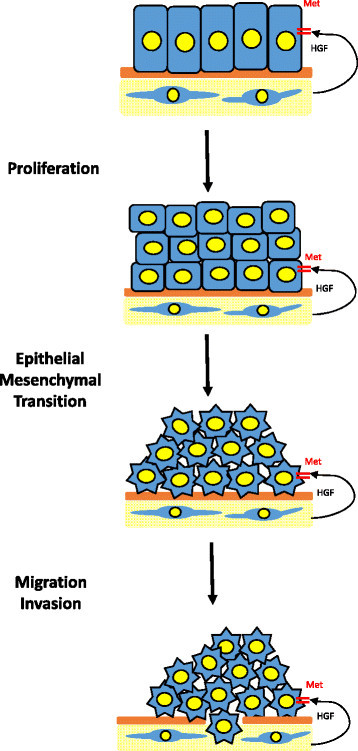
***Schematic representation of HGF/Met-mediated epithelial mesenchymal transition (EMT) and promotion of malignant progression.*** HGF-mediated Met activation and signaling can induced multiple pathways that are involved in stimulating cancer cell proliferation, survival, motility, angiogenesis, invasion and metastasis. Normal epithelial cells display a highly differentiated morphology characterized by a single layer of cells anchored by their basal lamina to the extracellular matrix. Aberrant Met activity will stimulate cell proliferation and EMT that ultimately results in changes in morphology and behavior, characteristic of a mesenchymal-like phenotype. EMT allows cancerous epithelial cells to become more mobile, invasive and metastatic in nature.

HGF-dependent Met activation in transformed epithelial cells promotes expression of a malignant mammary epithelial phenotype [8]. Met-induced tumor cell progression to a malignant phenotype is a multi-step process, characterized by the loss of epithelial polarity, loss of cell to cell adhesions, degradation of basal lamina, and increased cell migration and invasion [3],[4],[26]-[29]. An initial step in Met-induced malignant progression is EMT (Figure 3). EMT is essential for migration of tumor cells from the site of the primary tumor, invasion into surrounding tissues and entrance into the systemic circulation [6],[8],[11],[12],[14]-[18]. HGF-dependent activation of Met signaling shows a direct correlation with a reduction of cadherin-based adherens junctions, followed by loss of E-cadherin and cytokeratins 8/18 expression, and increased expression of mesenchymal proteins such as vimentin [6],[8],[11],[12],[14]-[18]. Epithelial cells that undergo EMT lose epithelial cell characteristics and acquire a mesenchymal phenotype, including changes in their morphology, increased motility and invasiveness [12].

**Figure 4:**
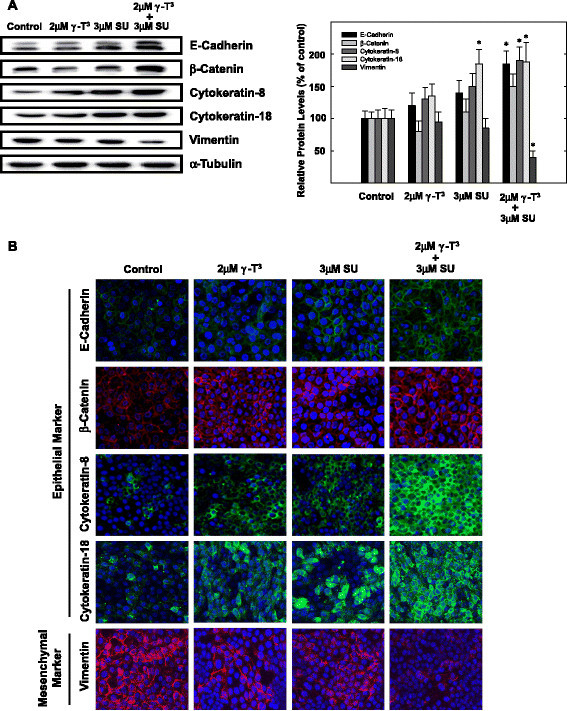
***Effects of γ-tocotrienol (γT***^***3***^***) and/or the Met tyrosine kinase inhibitor, SU11274 (SU), on the expression of major epithelial and mesenchymal cellular protein markers in + SA mammary tumor cells.*** Cells were incubated with control or treatment media containing subeffective doses of γ-tocotrienol (2 μM) and SU11274 (3 μM) either alone or in combination containing 10 ng/ml HGF as a mitogen for 3-days. **(A)** Afterwards, whole cell lysates were prepared and subjected to polyacrylamide gel electrophoresis and Western blot analysis for E-cadherin, β-catenin, cytokeratin-8, cytokeratin-18, and vimentin. α-Tubulin was visualized to ensure equal sample loading in each lane. Scanning densitometric analysis was performed for each blot to visualize the relative levels of proteins. Integrated optical density of each band was normalized with their corresponding α-tubulin and control treatment bands and shown in bar graphs. Vertical bars indicate the fold-change in protein levels in various treatment groups ± SEM as compared with their respective vehicle-treated control group. *P < 0.05 as compared to their respective vehicle-treated control group. **(B)** Treatment effects on immunocytochemical fluorescence staining of epithelial and mesenchymal markers in + SA mammary tumor cells after a 3-day culture period. +SA cells were seeded on 4-chamber culture slides at a density of 1x10^5^ cells/chamber (3 replicates/group) and allowed to attach overnight. Cells were then washed with PBS and incubated with vehicle control or treatment defined media containing 10 ng/ml HGF for 3 days in culture. At the end of treatments, cells were fixed with 4% formaldehyde/PBS and permeabilized with 0.2% triton X-100. Fixed cells were blocked and incubated with specific primary antibodies followed by incubation with Alexa Fluor 594- or 488-conjugated secondary antibodies. Red or green color indicates positive fluorescence staining for target proteins, while blue color represents nuclear counter staining with DAPI. Magnification is 200X. Figure obtained from reference 25 with permission.

Since combined treatment with low dose of γ-tocotrienol and SU11274 has profound inhibitory effects on tumor cell growth and HGF-dependent Met activation and signaling, additional studies aimed to determine effects of these agents on breast cancer cell epithelial and mesenchymal cell marker expression. Results show that neoplastic mammary epithelial cells grown in serum-free culture medium containing HGF as a mitogen display a strong transition from epithelial to mesenchymal cell phenotype, as evidenced by low epithelial marker and high mesenchymal marker protein expression [25]. Specifically, cells display relatively low levels of the epithelial cell protein markers E-cadherin, β-catenin, cytokeratin-8, and cytokeratin-18, and a relatively high levels of the mesenchymal protein marker, vimentin [25]. These studies also show that treatment with subeffective (non-growth inhibiting) dose of γ-tocotrienol or SU11274 alone causes only a slight reduction in EMT, characterized by a slight increase in the level of some epithelial markers (cytokeratin-8 and cytokeratin-18), but little or no change in vimentin expression [25]. However, combined treatment with these subeffective doses of γ-tocotrienol and SU11274 results in reversal of EMT, as demonstrated by changes in cell marker expression including augmentation of epithelial protein markers E-cadherin, β-catenin, cytokeratin-8, and cytokeratin-18, and corresponding reduction in mesenchymal protein marker (vimentin) expression [25]. Treatment effects on epithelial and mesenchymal cell marker expression in mammary cancer cells are shown in Figure 4. Taken together, these findings provide evidence that γ-tocotrienol inhibits HGF-dependent Met activation and signaling and causes reversal of EMT. However, additional studies are required to verify that these effects obtained *in vitro* occur in *in vivo*.

**Figure 5:**
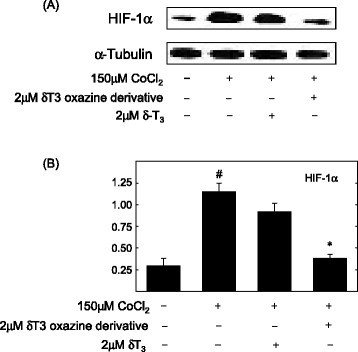
***γ-Tocotrienol and its oxazine derivative on CoCl***_***2***_***-induced hypoxia and HIF-1α expression in mammary tumor cells.*** *HIF-1*α is a hypoxia-inducible transcription factor that subsequently acts to stimulate blood vessel formation and promote survival of cancer cells during hypoxic conditions. **(A)** Effects of 150 μM CoCl_2_ (hypoxic, but not cytotoxic dose) alone and in combination with non-growth inhibitory doses (2 μM) of δ-tocotrienol or the δ-tocotrienol oxazine derivative, 12-((R)-6,8-dimethyl-8-((3E,7E)-4,8,12-trimethyltrideca-3,7,11-trienyl)-9,10-dihydrochromeno[5,6-e] [1],[3]oxazin-2(1H,3H,8H)-yl)dodecan-1-ol), on HIF-1α levels in + SA mammary tumor cells. Non-growth inhibitory doses of α-tocotrienol and its oxazine derivative were selected because high doses of these agents were found to initiate apoptosis and induce cancer cell death, which would confound the interpretation of the results regarding the effects of these agents on tumor cell compensatory response to CoCl_2_ hypoxia. +SA cells were seeded at concentration of 1.5X10^6^ in 100 mm culture dishes and allowed to attach overnight. The following day, cells were divided into groups and exposed to their respective treatments for a 24 hr incubation period. Afterwards, whole cell lysates were prepared for Western blot analysis. **(B)** Scanning densitometric analysis was performed on all blots done in triplicate and the integrated optical density of each band was normalized with corresponding α-tubulin, as shown in the bar graphs below their respective Western blot image. Vertical bars indicate the normalized integrated optical density of bands visualized in each lane ± SEM. #*P* < 0.05 compared to the vehicle-treated control group. *P < 0.05 as compared to the hypoxic group treated with CoCl_2_ alone. This figure was obtained from reference 65 with permission.

Met expression is also responsive to various types of stress, particularly low oxygen tension or hypoxia [22]. Rapidly-growing solid tumors often display insufficient blood flow and oxygen deficiency within deep inner regions of the tumor. In response to hypoxic conditions, tumor cells are able to adapt by altering metabolism and phenotypic characteristics [20],[58]. This compensatory response to hypoxia is mediated by increased production of hypoxia-inducible factor-1 (HIF-1), a transcription factor that promotes cell survival during hypoxic conditions [21],[59]-[61]. HIF-1 is a heterodimer consisting of HIF-1α and HIF-1α subunits [21]. HIF-1α is constitutively expressed in most cells, whereas HIF-1α is inducible and is characteristically over-expressed in cancer cells during hypoxic conditions [21]. Overexpression of HIF-1α is also associated with elevated production of VEGF, a growth factor that promotes tumor angiogenesis, invasion and metastasis [62]. Studies show that during hypoxia, Met expression is increased through binding of HIF-1 to the Met promoter region to amplify HGF-dependent Met activation and signaling, facilitating cancer cell malignant progression [22]. Recent studies show that during hypoxic conditions, natural and semisynthetic derivatives of tocotrienols inhibit mammary tumor cell expression of HIF-1, Akt/mTOR activity and VEGF production *in vitro* and *in vivo*[63]. Although these findings suggest that tocotrienol treatment can also act to suppress the compensatory mechanisms that promote tumor growth and survival during hypoxic conditions, possibly through the inhibition of HGF/Met activation, additional studies are required to establish a direct relationship. The effects of γ-tocotrienol and its oxazine derivation on HIF-1α expression in mammary tumor cells during CoCl_2_-induced hypoxia are shown in Figure 5.

## Conclusion

It has become increasingly evident during the past decade that HGF-dependent Met activation and signaling plays a major role in malignant progression and metastasis. As a result, substantial interest has focused on developing therapeutic strategies that target the HGF/Met axis. A variety of emerging approaches are directed toward one or more of the components within this signaling pathway. Although the majority of research conducted in this area has focused on preclinical studies using cell culture and animal experimental models, a great number of therapeutic agents (onartuzumab, rilotumumab, tivantinib, and cabozantinib) developed and refined in preclinical experiments are increasingly the subject of clinical trials. Therapies that disrupt cancer cell proliferation, survival, motility, angiogenesis, invasion and metastasis will provide great benefits in the treatment and cure of cancers that display aberrant Met activity. Recent evidence suggest that γ-tocotrienol may also provide some benefit in the treatment of breast cancers that displays Met dysregulation.

## Author contribution

The corresponding author was responsible for organizing, writing and preparing the illustrations presented in this article.

## Author information

Dr. Paul W. Sylvester is the Pfizer, Inc. - B. J. Robison Endowed Professor in Pharmacy in the School of Pharmacy at the University of Louisiana at Monroe, Monroe, LA. Dr. Sylvester received his B.S. degree in Biology from Western Michigan University, Kalamazoo, and his Ph.D. in Physiology from Michigan State University in East Lansing, and completed his postdoctoral training in Experimental Therapeutics at Roswell Park Cancer Institute in Buffalo, NY. Dr. Sylvester's research interests include examining the relationship of nutrition and cancer, with particular emphasis on understanding the intracellular mechanisms mediating the anticancer effects of tocotrienols. Dr. Sylvester is a member of the American Association for Cancer Research since 1986.

## Authors’ original submitted files for images

Below are the links to the authors’ original submitted files for images. Authors’ original file for figure 1 Authors’ original file for figure 2 Authors’ original file for figure 3 Authors’ original file for figure 4 Authors’ original file for figure 5

## Abbreviations

Akt: Protein kinase B
EGF: Epidermal growth factor
EGFR/ErbB: Epidermal growth factor receptor
EMT: Epithelial-mesenchymal transition
HER: Human epidermal growth factor receptor
HGF: Hepatocyte growth factor
HIF-1: Hypoxia-inducible factor-1
MAPK: Mitogen-activated protein kinase
Met: Mesenchymal epithelial transition factor receptor
mTOR: Mammalian target of rapamycin
NFκB: Nuclear factor kappa-light chain-enhancer of activated B cells
PI3K: Phosphoinositide 3-kinase
siRNA: Small interfering RNA or silencing RNA
STAT: Signal transducer and activator of transcription
VEGF: Vascular endothelial growth factor
γT_3_: γ-tocotrienol
δT_3_: δ-tocotrienol
