# Supplementary figures and images for: Targeting met mediated epithelial-mesenchymal transition in the treatment of breast cancer
Source: Clin Transl Med. 2014 Sep 26;3:30. doi: 10.1186/s40169-014-0030-5 (PMC4883993; doi:10.1186/s40169-014-0030-5)

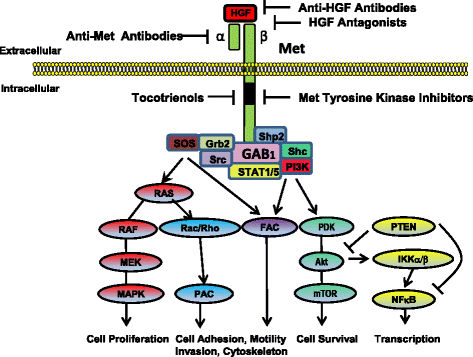

Supplement: Supplementary file 1 — Authors’ original file for figure 1 [file 40169_2014_30_MOESM1_ESM.gif]

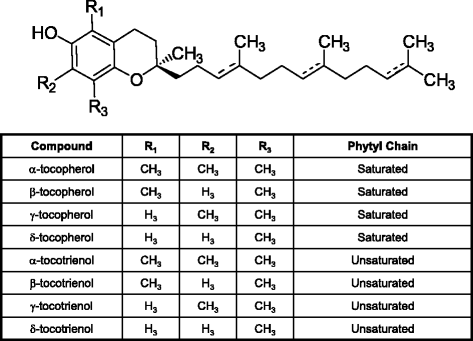

Supplement: Supplementary file 2 — Authors’ original file for figure 2 [file 40169_2014_30_MOESM2_ESM.gif]

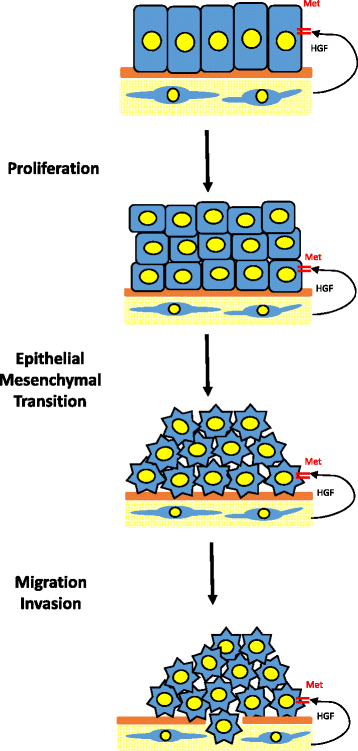

Supplement: Supplementary file 3 — Authors’ original file for figure 3 [file 40169_2014_30_MOESM3_ESM.gif]

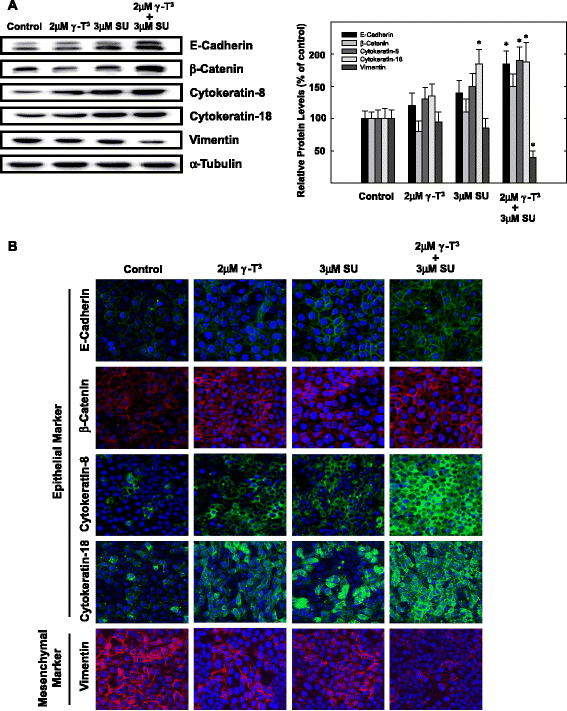

Supplement: Supplementary file 4 — Authors’ original file for figure 4 [file 40169_2014_30_MOESM4_ESM.gif]

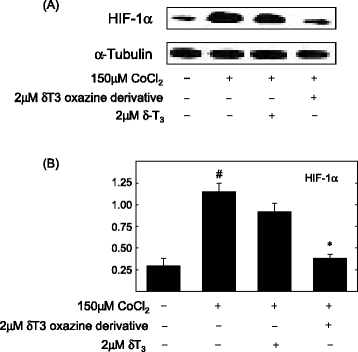

Supplement: Supplementary file 5 — Authors’ original file for figure 5 [file 40169_2014_30_MOESM5_ESM.gif]
